# Reliability Study of Metal-Oxide Semiconductors in Integrated Circuits

**DOI:** 10.3390/mi15050561

**Published:** 2024-04-24

**Authors:** Boris V. Malozyomov, Nikita V. Martyushev, Natalia Nikolaevna Bryukhanova, Viktor V. Kondratiev, Roman V. Kononenko, Pavel P. Pavlov, Victoria V. Romanova, Yuliya I. Karlina

**Affiliations:** 1 Department of Electrotechnical Complexes, Novosibirsk State Technical University, 20, Karla Marksa Ave., 630073 Novosibirsk, Russia; 2 Department of Advanced Technologies, Tomsk Polytechnic University, 634050 Tomsk, Russia; 3 Laboratory of Geochemistry of Ore Formation and Geochemical Methods of Prospecting, A. P. Vinogradov Institute of Geochemistry of the Siberian Branch of the Russian Academy of Sciences, 664033 Irkutsk, Russia; nnb@igc.irk.ru (N.N.B.); v.kondratiev@igc.irk.ru (V.V.K.); 4 Computer Hardware and Software Laboratory, Institute of Information Technologies and Data Analysis, Irkutsk National Research Technical University, 664074 Irkutsk, Russia; iistu_politeh@mail.ru; 5 Department of Electrical Complexes and Systems, Kazan State Power Engineering University, 420066 Kazan, Russia; pavlov2510@mail.ru; 6 Department of Energy, Trans-Baikal State University, 672039 Chita, Russia; romanova181@mail.ru; 7 Stroytest Research and Testing Centre, Moscow State University of Civil Engineering, 26, Yaroslavskoye Shosse, 129337 Moscow, Russia; jul.karlina@gmail.com

**Keywords:** metal-oxide semiconductors, microcircuit, reliability, rejection tests, temperature regime, activation energy

## Abstract

This paper is devoted to the study of CMOS IC parameter degradation during reliability testing. The paper presents a review of literature data on the issue of the reliability of semiconductor devices and integrated circuits and the types of failures leading to the degradation of IC parameters. It describes the tests carried out on the reliability of controlled parameters of integrated circuit TPS54332, such as quiescent current, quiescent current in standby mode, resistance of the open key, and instability of the set output voltage in the whole range of input voltages and in the whole range of load currents. The calculated values of activation energies and acceleration coefficients for different test temperature regimes are given. As a result of the work done, sample rejection tests have been carried out on the TPS54332 IC under study. Experimental fail-safe tests were carried out, with subsequent analysis of the chip samples by the controlled parameter quiescent current. On the basis of the obtained experimental values, the values of activation energy and acceleration coefficient at different temperature regimes were calculated. The dependencies of activation energy and acceleration coefficient on temperature were plotted, which show that activation energy linearly increases with increasing temperature, while the acceleration coefficient, on the contrary, decreases. It was also found that the value of the calculated activation energy of the chip is 0.1 eV less than the standard value of the activation energy.

## 1. Introduction

The development of microelectronics is caused by the constant growth of the degree of integration of integrated circuits, which in turn leads to an increase in the occurrence of failures of the element base of REA and a decrease in its reliability [1,2]. Ensuring the quality and reliability of ICs is of particular importance since the characteristics of these products largely determine the tactical and technical characteristics of weapon systems. The pace of microelectronics development significantly complicates the task of predicting and assessing the quality of ICs [3,4]. The requirements for the reliability of ICs are constantly increasing, as the requirements for the basic materials for the manufacture of microcircuits are currently tightened. Failures can occur during rejection tests, electrical testing, transport, storage, and application [5]. At the same time, the design and technological design of microcircuits has been changing. All of this affects the physical and technical processes, the activation energy, and the nature of failures [6,7].

All of this makes it necessary to carry out studies to determine activation energy, failure mechanisms for microcircuits, current production, and methods of accelerated testing for failure-free operation and time to failure [8,9].

The problem of cumulative degradation of integrated circuits is quite acute, and it is important to determine the reasons for the deterioration of the parameters of integrated circuits and methods for increasing the reliability of circuits, both physical and technological. Diagnosing integrated circuits and testing them makes it possible to identify the reasons responsible for the deterioration of parameters, which is undoubtedly a key point in increasing the reliability of electronic equipment. Naturally, direct diagnostic methods and tools, based on a set of studies of the parameters of the integrated circuit (IC) itself, are more attractive since they directly allow one to judge the reliability of this circuit. However, not all reasons can be unambiguously established on the basis of direct methods only, so the search, development, and improvement of indirect methods with the use of additional tools and methods for diagnosing materials, technological processes, and operations is no less important. Determining the causes of failures and deterioration of IC parameters makes it possible to better monitor the process of rejecting low-quality circuits, as well as to develop new methods for increasing the reliability of ICs, relying on existing physical and technological methods. In this case, it is absolutely necessary to take into account the physical processes occurring in ICs, especially those based on composite materials.

The relevance of this work is to determine and ensure the reliable characteristics of the IC, which is the most important condition when designing microcircuits [10,11].

In the process of the operation of industrial products, due to wear and tear and irreversible aging processes, the characteristics of the hardware will change [12]. One of the key complex properties of products is reliability, which is defined as the property of the object to preserve in time within the established limits the values of all parameters characterising the ability to perform the required functions in the specified modes and conditions of application, maintenance, repairs, storage, and operation [13,14].

The purpose of this paper is to investigate and subsequently analyse the results of reliability tests on controlled parameters of a step-down pulse voltage converter chip with integrated power key and galvanic isolation.

For this purpose, the following objectives were set:

- Investigation of the possible types and causes of IC failures;
- Carrying out experimental tests for failure-free operation of the microcircuit;
- Analyses of the results of tests on reliability, MTBF, and electro-thermo training of ICs.
- Determination of the activation energy of failure mechanisms of IMCs;
- Calculation of the acceleration factor of the chip.

### 1.1. Causes of IC Failures

Functional parameters of microcircuits are the main criteria for their quality, and their consistency over time determines the operational reliability of the integrated circuit. Therefore, the control of functional parameters of microcircuits is one of the most important technological stages in the production of microcircuits. The causes of failures were investigated in [15,16], and the operational reliability parameters affecting the integrated circuit were determined.

At the same time, issues related to the reliability of integrated circuits require compromise solutions. Namely, the increasing complexity of functions requires a large number of elements and components in the structure of integrated circuits, which worsens their reliability [17]. The efficiency of process control and quality control of integrated circuits is reduced due to the decrease in the completeness of verification due to a significant increase in the sets of combinations of input signals during testing, which provides a complete and reliable assessment of the quality of their functioning under conditions of ever-increasing labor intensity of the control [18,19]. As a result of the analysis of IC failures, it was determined that most of the reduction in yields is due to technological factors.

### 1.2. Technological Factors Affecting IC Failures

**Figure 1:**
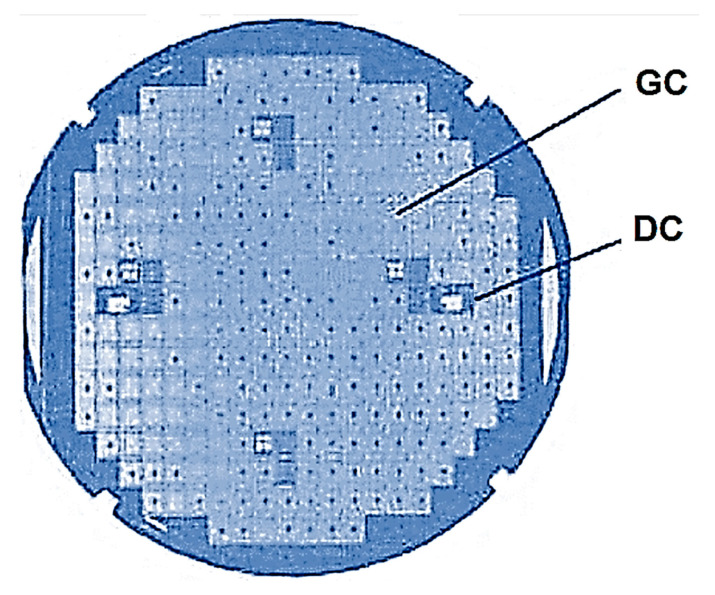
Photograph of the plate showing the areas of high and low yields of good crystals: GC, good crystal; DC, rejected crystal.

On the plate subjected to control experiments, areas with a high percentage of yearly crystals, areas with a low percentage of such crystals, and sometimes areas with no yearly crystals at all are highlighted (Figure 1).

It was found that the areas with low yields are caused by technological factors. At the same time, the authors highlighted the main technological factors affecting IC failures:

- deviations from the specified values of oxide and polycrystalline silicon layer thicknesses;
- deviations of the resistances of the implanted layers;
- errors of geometrical dimensions of elements at the lithographic formation of circuit topology;
- errors of phototemplate matching at different stages of the technological process.

Some of these factors are interrelated. For example, if the wafer etch time is chosen based on a polycrystalline silicon layer thickness that is greater than the average value, then the polycrystalline silicon layer thickness is less than the average value, which means that the stripping depth is too high [20,21]. The gates of MOS transistors have smaller dimensions in regions with smaller polycrystalline silicon layer thickness. This fact leads to a too-small channel length in the MOS transistor; as a result, the transistors do not switch off when the appropriate voltage is applied to the gate electrode. Because of this, the functioning of circuits may be disturbed, or leakage currents may increase excessively [22].

Deviation in the doping level of the implanted layers can lead to changes in contact resistances and deviation in the thickness of dielectric layers can lead to changes in the dimensions of contact windows [23]. These factors can cause failures of circuit operation in the presence of tracks, whose characteristics are determined by the value of contact resistance [24].

With improvements in processes and operations, the effects of many of these circumstances that limit circuit yields can be reduced or eliminated, but new causes of circuit failures are possible [25,26].

Certain areas on the wafer can have a low yield percentage not only because of exceeding the established permissible deviations of IC parameters associated with violations of manufacturing technology but also due to the fact that the design of the chip does not take into account the impact of possible deviations of IC parameters and the relationship between the deviations of various parameters [27].

Not all faults are failures. Faults that do not lead to failure are called defects [28,29]. Depending on the nature and level of factors affecting the integrated circuit, different types of tests have different effectiveness in terms of identifying hidden defects that determine the reliability of products.

The leading defect that causes the possibility of a chemical or electrochemical reaction is a leaky enclosure. Moisture penetrates through the holes into the housing and causes reactions. Analysis of failed ICs shows that the presence of holes in the cases is more dangerous at the final stages of the manufacturing process than during actual operation under normal conditions [30,31,32,33,34].

**Figure 2:**
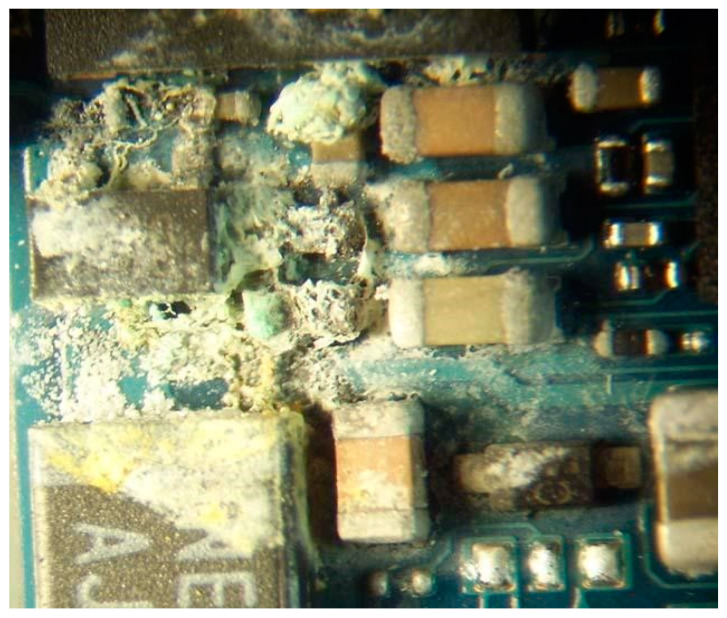
Electrochemical corrosion of chip leads.

Figure 2 shows the traces of electrical corrosion of the chip leads revealed by the experiments.

Multi-component alloys are used in the production of ICs. Electrolytic corrosion occurs when there is a potential difference between two neighbouring electrodes, and a sufficient amount of moisture is deposited from the surrounding area. The metal of the dissolving anode is then gradually transported to the cathode, leaving a conductive path along the way. When the metallisation area is completely dissolved, an interruption in the circuit occurs [35,36,37,38].

Dislocations can develop in the substrate single crystal during the operation of microcircuits. If dislocations cross *p-n junctions*, it leads to a sharp increase in leakage of these junctions [39,40,41,42].

## 2. Materials and Methods of Chip Reliability Testing

The general plan of the experiment was as follows: measurements take place on the developed measuring stand, and the calculation of instability coefficients of a given output voltage is carried out. Measurements of the parameters were carried out during the acceptance of the microcircuit and preparation of samples for external influencing factors under normal conditions, as well as at reduced and increased temperatures. Then, rejection and failure tests and determination of the activation energy of the IC were carried out.

### 2.1. Description of the Object of Research

The object of the test is a TPS54332 (Texas Instruments, Dallas, TX, USA) chip, which is a DC-DC voltage converter acting as a step-down pulse voltage converter with an integrated power key and galvanic isolation and an output current limit up to 0.55 A.

Voltage converter integrated circuits used in switching power supplies are an integral part of radioelectronic systems that are important for powering devices [43]. The need for the development of ICs is conditioned by reliability, import substitution, trends in improving technical characteristics (speed, performance, reduction of power consumption, increased resistance to special effects, etc.), and miniaturisation of developed products for various purposes [44].

**Figure 3:**
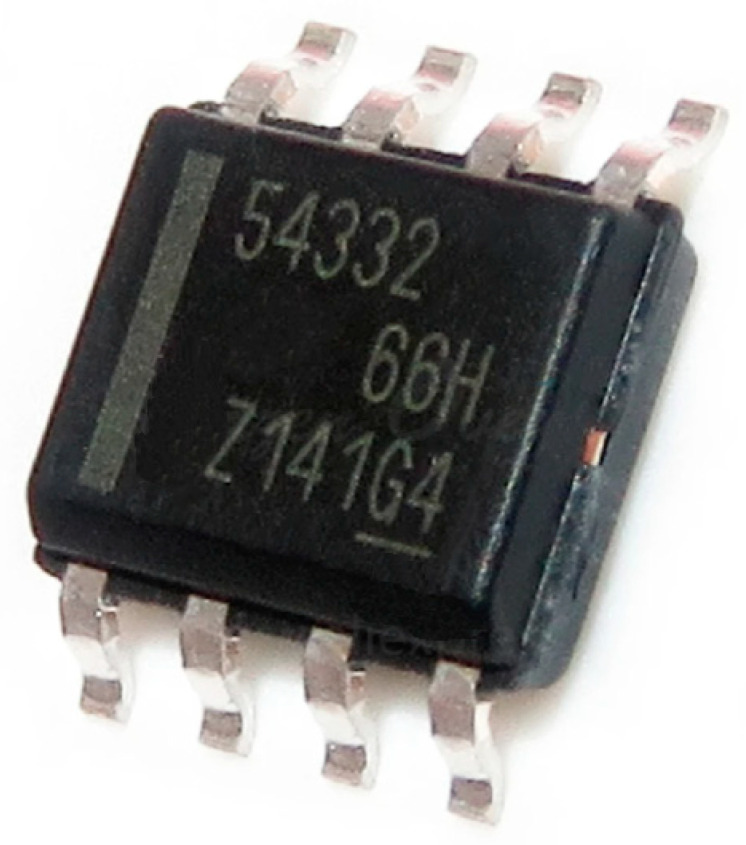
Functional diagram of DC-DC voltage converter.

The functional diagram of the DC-DC voltage converter is shown in Figure 3. The peculiarity of the IC’s circuit diagram is that it has two functional parts—digital and analogue, —so the functional diagram has a ground pin to the digital and analogue parts [45].

TPS5432 microcircuit is designed for use as a secondary power supply with output voltage from 1.5 to 6 V (depending on the inclusion scheme) and input supply voltage in the range of 10–33 V. At the same time, the microcircuit is also intended for use with galvanic isolation, where a low-power transformer is used as an isolation element.

The design of the step-down pulse voltage converter includes the following functional blocks:

- internal power supply voltage generation unit;
- PWM control unit;
- power key;
- power key driver;
- thermal protection unit;
- logic control unit;
- feedback control unit.

A distinctive feature of the chip is the relatively high value of its output current. The maximum load current of the TPS54332 chip is 0.55 A.

The main purpose of the chip is to provide uninterrupted power supply to consumers, which can be both chips and various kinds of switches, including mechanical ones.

The application of the chip provides:

- Increased life cycle of advanced special-purpose vehicles by increasing the time until failure of the developed ICs and the life expectancy;
- high level of resistance to the effects of UHF;
- Reduction of mass-dimension indicators of REA power supplies.

Based on the applicability of the chip, the electrical parameters of the DC-DC voltage converter are set in the technical specifications (TS) for development, which are presented in Table 1, as at acceptance and delivery.

### 2.2. Description of Installations

The measurement of electrical parameters is carried out under normal conditions and extreme operating temperatures:

1. Normal conditions: 25 ± 10 °C.
2. Temperature limits: −(60 ± 3), (85 ± 3) °C.

Measurements of electrical parameters under normal conditions are carried out at the measuring bench installation. The working place of the measuring bench is designed for the connection of external components to the tested chip, configuration of the required measurement circuits, and measurement of electrical parameters of the DC-DC step-down voltage converter chip (TPS54332) in the H08.24-2V package under normal conditions (25 ± 10 °C) and extreme operating temperatures −(60 ± 3), (85 ± 3) °C) with the help of external standard devices.

The measuring stand is designed for operation at temperatures from +10 to +35 °C, relative humidity from 65 to 80%, and atmospheric pressure from 86 to 106 kPa (630 to 800 mmHg).

The measuring bench allows for measuring the electrical parameters of microcircuits in the temperature range ((25 ± 10), −(60 ± 3), (85 ± 3) °C) using passive thermostat 55.34083.803 EGO.

**Figure 4:**
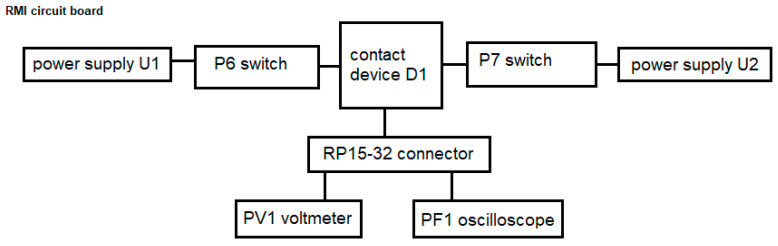
Structural diagram of the measuring bench equipment.

Figure 4 shows the measurement connection diagram of the measuring bench equipment, which includes the RMI board, switches P6 and P7, power supplies U1 and U2, contacting device D1, connector RP15-32, oscilloscope PF1, voltmeter PV1.

The measuring bench allows for measuring the following electrical parameters in manual mode:

- limit output current, Iout_m1;
- frequency of the internal oscillator, F;
- instability of the set output voltage over the entire input voltage range, *K_u_*;
- instability of the set output voltage over the whole range of load currents, *K_I_*.

### 2.3. Methodology of Measurements on the Measuring Bench and Calculation of Instability Coefficients of the Set Output Voltage

When measuring the parameters I_v__m^2^, *K_U_* and *K_I_*, F, it is necessary to program the sources U1 and U2 to voltages of 10 V and 24 V, respectively. Measure with a PV1 voltmeter the output voltage at the minimum output current (U_vh_Imin_); it should be in the range from 4.7 to 5.4 V in the whole temperature range ((25 ± 10), −(60 ± 3), (85 ± 3) °C).

Measure the output voltage at the maximum output current (U_v_Imax_); it should not be less than 4.7 V in the whole temperature range, taking into account the load R_n_ = 8.6 Ohm at normal temperature (25 ± 10 °C) (R_n_ = 13.6 Ohm at temperatures −60 ± 3, 85 ± 3 °C).

Calculate the parameter *K**_I_*** using the formula:

(1) $$K_{I}=U_{out}I_{\max}\;-\;U_{out}I_{\min}.$$

In all measurements, the frequency of the internal oscillator F must be in the range from 160 to 360 kHz at 25 °C and from 100 to 400 kHz at temperatures −60 and 85 °C.

Program the source U_2_ for voltage 10 V. Measure with a PV1 voltmeter the output voltage at minimum input voltage (U_vh__U_vh.min_); it should be in the range from 4.7 to 5.4 V in the whole temperature range.

Program the source U2 for voltage 33 V. Measure the output voltage at the maximum input voltage (*U_out_*_U_inp.max_) with the PV1 voltmeter; it should be in the range from 4.7 to 5.4 V in the whole temperature range.

Calculate the parameter *K_U_* using the formula:

(2) $$K_{U}=U_{\mathrm{out}-}U_{\max}\;-\;U_{\mathrm{out}-}U_{\min},$$

Measurements of the parameters were carried out during the acceptance of the chip and the preparation of samples for external influencing factors under normal conditions, as well as at reduced and elevated temperatures.

### 2.4. Test Methodology in the Heat and Cold Chamber

When testing semiconductor products at elevated temperatures, the ERSTEVAK EVCLIM-KTCHV-408 K climatic heat-cold-humidity chamber is used [46].

The unit has the following technical characteristics:

- The temperature range in the chamber’s usable volume is 45 to 125 °C.
- The error of temperature setting and maintenance, taking into account the uneven distribution in the usable volume, does not exceed ±3 °C.
- Loading of the chamber with semiconductor devices is cassette.
- The maximum electrical power consumed by the chamber during the mode setting period is not more than 6 kW.
- The camera provides continuous operation for 10,000 h with 128 kbyte archive memory and data display on the alphanumeric display of the temperature controller.

The operation of the chamber is based on the principle of creating and maintaining a temperature regime at a given level [47].

When testing semiconductor products at subzero temperatures, use a chamber in cold mode.

Installation Characteristics:

- Operating temperature of the chamber −(5 ÷ 60) °C.
- Temperature maintenance accuracy ±1 °C-/in the instrument test area.
- The time to reduce the chamber temperature from +20 to −60 °C is 30 min.

Loading the chamber with semiconductor probes is carried out using cassettes.

Liquid nitrogen is used as a cooling agent. The consumption of liquid nitrogen is 7 kg per 2.5 h.

## 3. Rejection Tests

A common way to improve the quality and reliability of a manufactured batch of semiconductor devices and integrated circuits is to carry out rejection tests or ETT (electro-termination) at the stage of output control at the manufacturing plant. Rejection tests are those tests that are performed at the production stage in order to identify and remove defective products [48,49].

**Figure 5:**
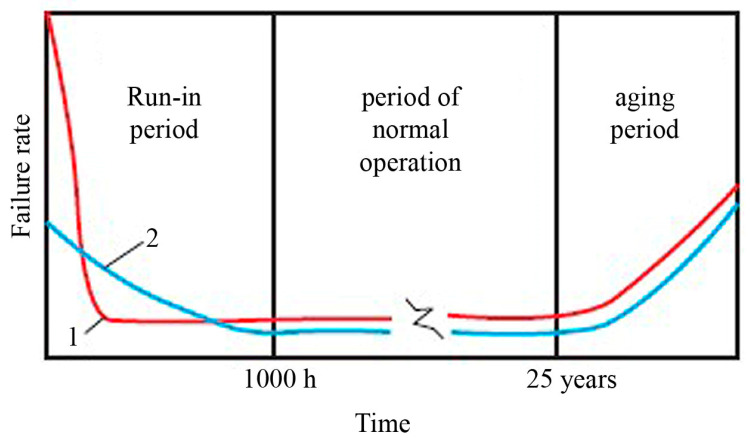
Typical dependence of semiconductor product failure rate on time: 1—without rejection tests; 2—with rejection tests performed.

It is assumed that there are no random failures of semiconductor products; each failure has a cause and is a consequence of the application of some load. “Weak” semiconductor products that remain undetected at the start of operation can cause failures of radio-electronic equipment (REA). For rejection testing to be effective, it is necessary to know what the loads are and how they accelerate the occurrence of failures. The experience of using integrated circuits in REA shows that the introduction of rejection tests [50] significantly increases the average level of their reliability (Figure 5).

Most semiconductor product failure mechanisms are accelerated by temperature and voltage or current, so during training, products should be operated at the maximum allowable voltage and maximum possible temperature [51]. However, at this temperature, there should be no thermal overload, logic state changes, or unacceptably high current densities in the metallisation. Thermal overload must be avoided because otherwise, the semiconductor junction temperature cannot be controlled, leading to rapid product failure [52,53].

Electro-thermal testing (ETT) is recognised as an effective means of accelerating operational failure mechanisms. It provides a lot of information in a short time, but reliable results can be obtained only on the basis of proper selection of electrical and thermal loads, identification of failure types and mechanisms corresponding to real operating conditions, and static processing of the obtained results [54].

The temperatures at which training is carried out are 65, 70, 85, 100, 125, and 150 °C.

ETT is carried out on special stands under strict temperature control. The difference in the failure rate of devices in hermetic and plastic cases is explained by a greater number of cases of gate breakdown in the latter, which are more sensitive to static charges than devices in hermetic metal cases [55].

Currently, the duration of ETT of different schemes in different modes is 48, 72, 96, 150, 168, 240 h, and in some cases, up to 1000 h. The temperatures at which training is carried out are 70, 85, 100, 125, and 150 °C.

In the course of work, rejection tests were carried out, followed by the measurement of electrical parameters of the investigated microcircuit and identification of unserviceable products. In total, a large number of measurements (about 700) were carried out.

**Figure 6:**
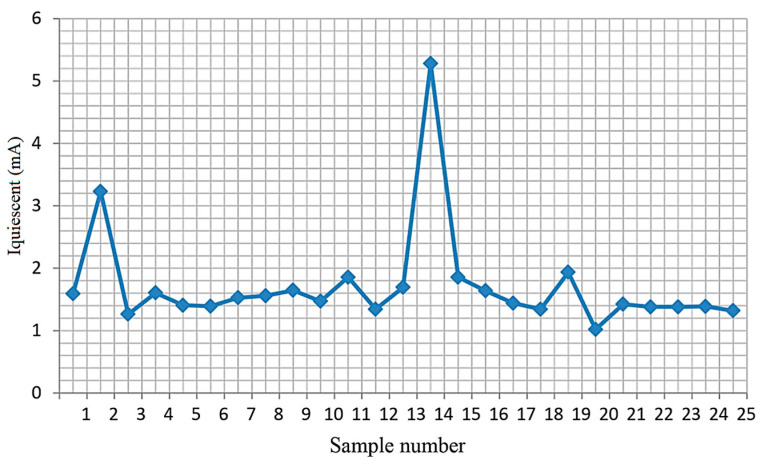
Quiescent current values at T = 25 °C.

**Figure 7:**
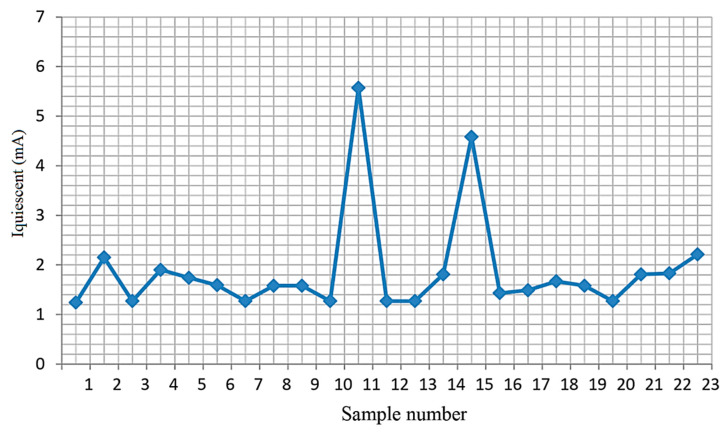
Quiescent current values at T = 85 °C.

**Figure 8:**
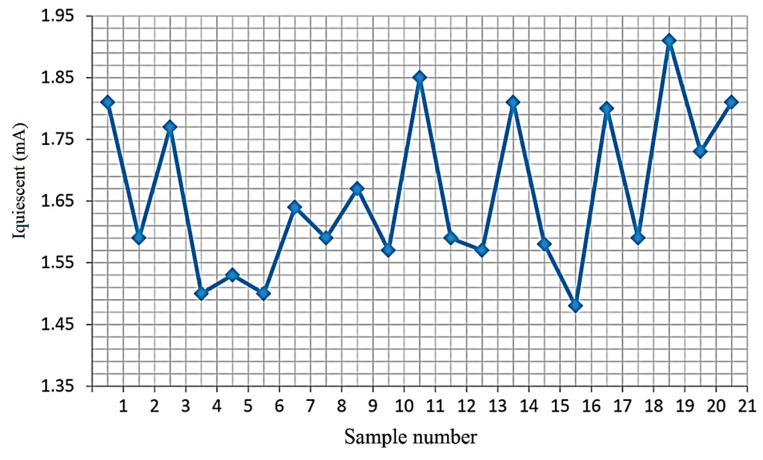
Quiescent current values at T = −60 °C.

Figure 6, Figure 7 and Figure 8 show the measured dependencies of the monitored quiescent current parameter of the TPS54332 chip.

Figure 6 shows the quiescent current values for 25 microcircuits at T = 25 °C. The graph shows that the samples numbered 2 and 14 are unsuitable for further operation. In these samples, the quiescent current is close to the limit of the permissible norm (the norm is 5.4 mA).

Figure 7 shows the quiescent current values for 23 microcircuits at T = 85 °C. The graph shows that the quiescent current value is as close as possible to the limit of the permissible norm (norm 9 mA) for two samples numbered 11 and 15.

Figure 8 shows the quiescent current values for 21 chips at T = −60 °C. The graph shows that all samples are within the permissible limit (9 mA). No failures were detected.

In the case of ETT, the rejection rate does not exceed the value set in the general specifications [56].

## 4. Failure Tests

Microcircuit failures are caused by changes in materials and structures as a result of degradation processes of different natures: electrical, radiation, thermal, and mechanical [57]. Each type of degradation process can be responsible for the occurrence of different types of failures. Failures can be categorised into the following types: crystal-related; in leads and interconnects; sealing-related; and caused by external conditions and overvoltages [58]. First of all, all failures are related to materials, process conditions, and operations.

The nature of flow and the manifestation of these failures depends on the specifics of technology and is determined by the nominal operating modes of the circuit [59]. In this regard, it is necessary to analyse the results of chip tests for failure-free operation and MTBF and electro-thermal training of the microcircuit under study in order to determine the possible types and causes of failures. Therefore, it is necessary to consider the possible methods of accelerated and experimental tests and to determine the methods of conducting studies of ICs [18]. Failure control tests include short-term and long-term failure tests [5].

Short-term tests are carried out in order to control the stability of the technological process of manufacturing products and to assess the compliance of the failure rate with the established requirements based on the generalisation of test results. Long-term tests are carried out to confirm the failure rate directly on the basis of test results or on the basis of generalisation of test results [23].

In order to carry out failure tests, accelerated tests are used—these are tests, methods, and conditions which will provide the necessary amount of information in a shorter period of time than in the conditions and modes of operation. Accelerated tests are subdivided into shortened and forced.

Abbreviated tests are those accelerated tests that take place without intensifying the processes that cause failure or damage. In abbreviated tests, in order to reduce the time required to obtain reliability indices, it is necessary to predict the behavior of the test object for a period longer than the duration of the test. Accelerated tests, on the other hand, are accelerated tests that are based on the intensification of processes that cause failure or damage. In accelerated tests, a special increase in the rate of loss of serviceability of the product is carried out.

Accelerated tests are used in order to reduce the time required to perform tests as compared to normal tests, i.e., tests whose methods and conditions will ensure that the required amount of information is obtained within the same period of time as the conditions and modes of operation stipulated in the normative technical documents for a given product. [18,19]

Selected results of measurements of electrical parameters after accelerated failure tests are given in Table 2. The quiescent current is taken as the controlled parameter.

**Figure 9:**
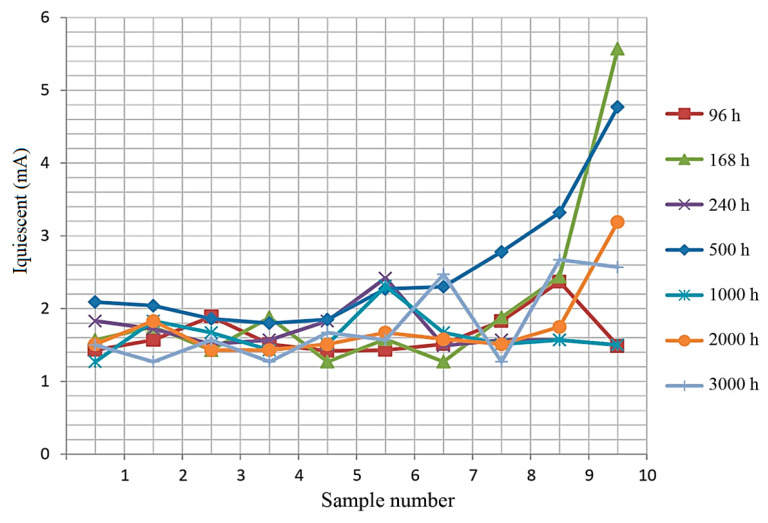
Quiescent current of 10 samples at different holding times.

Figure 9 shows the results of quiescent current measurements for samples selected from the total number of chips (10 samples) for different dwell times at T = 85 °C.

It can be seen from Figure 9 that the quiescent current readings are within acceptable limits (9 mA is the norm).

The number of failures at the time of verification after long-term tests (3000 h dwell time) is 11 out of 135 pcs.

In addition to measurements of quiescent current, the reliability study included additional measurements of the following parameters: quiescent current in standby mode and voltage at maximum and minimum currents (I_min_ = 500 mA, I_max_ = 3.3 A) and at maximum and minimum input voltage (U_min_ = 10 V, U_max_ = 33 V), the values of which are necessary to calculate the coefficients of instability of the specified output voltage over the entire range of input voltage and the entire range of load currents.

The values of output current, open key resistance, and instability coefficients of the set output voltage over the entire input voltage range and over the entire load current range were also calculated. Rejection was carried out according to the controlled parameter quiescent current I_quiescent_.

## 5. Determination of the Activation Energy of IMC

The reliability of integrated circuits depends entirely on the degradation processes occurring in the product and leading it to a failure state. They are characterised by the average rate ν_1_ and activation energy *E_aj_*. The rate of many chemical reactions and physical processes is determined by the Arrhenius Equation (3) [20].

(3) υ=qexp(−ΔE/kT),

where *q* is the proportionality coefficient determining the intensity of the reaction, i.e., the frequency of interaction acts in it; Δ*E* is the activation energy, which determines the barrier of different states in the reaction, eV; *k* is the Boltzmann constant (8.617 × 10^−5^ eV/˚K); *T* is the temperature, ˚K.

When, during a physical or chemical process, an atom, molecule, or ion of an IMU component material transitions from one state to another by overcoming Δ*E*, the probability that this transition occurs due to the thermal energy of the IMU material is proportional to the value exp(−Δ*E*/*kT*).

Drawing an analogy between chemical reactions and IMU degradation processes, we can write in Formula (4):

(4) $$v_{1}=A\exp(-E_{aj}/kT),$$

where *ν*_1_—average rate of degradation process; *E_aj_*—activation energy of degradation process; *A*—proportionality coefficient, which in the real temperature range from the application mode to the test mode is assumed to be constant.

To determine the activation energy based on IMU failures, we will use Formula (5).

(5) $$E_{a}=8.62\cdot 10^{-2}\beta_{1},$$

where *β*_1_ is the regression coefficient characterising the slope of the regression line, determined by Formula (6).

(6) $$\beta_{1}:=\frac{\sum_{z=1}^{S}(\mu_{z}\cdot x_{z})-\frac{1}{S}\cdot \left(\sum_{z=1}^{S}\mu_{z}\right)\cdot \left(\sum_{z=1}^{S}x_{z}\right)}{\left(\sum_{z=1}^{S}{x_{z}}^{2}\right)-\frac{1}{S}\cdot \left(\sum_{z=1}^{S}x_{z}\right)},$$

where *Z*—test mode number; *S*—total number of test modes; *T*_trans.z_ and *Q*_trans.z_—transition temperature in degrees Celsius and Kelvin, respectively (*Q*_trans.z_ = *T*_trans.z_ + 273); *d_Z_*—number of failures, pcs; *t_iz_*—time to failure of the *i*-th IMC in Z-mode, h; *y_iz_* = ln(*t_iz_*_)_—dependent variable (function of time); $x_{z}=\frac{10^{3}}{Q_{\mathrm{trans}.z}}$—independent variable (function of mode); $\mu :=\frac{\sum_{i=1}^{d_{z}}y_{iz}}{d_{z}}$—mathematical expectation of the logarithm of time to failure.

Based on the test results, the activation energy values for 2–3 µm design ICs at different temperatures (65, 85, 115, 125) °C were calculated using Mathcad 15.0 software and are presented in Table 3.

To predict the durability of the TPS54332 integrated circuit, a method has been proposed to conduct accelerated testing of the TPS54332 under higher load conditions. The aging process accelerates, and degradation of parameters occurs in the same way as in normal operation. The results obtained are extrapolated to normal operating conditions. This makes it possible to study the “aging” period of an integrated circuit in a relatively short period of time through the relationship of failure mechanisms with the time of their manifestation. This method was proposed due to the fact that increasing temperature and electric field strength accelerate the aging process of the microcircuit. The role of failure mechanisms in this process, including degradation ones, is associated with physicochemical reactions of the microcircuit structure.

The main characteristic of accelerated tests is the acceleration factor. The acceleration factor is a number that indicates how many times the duration of accelerated tests is shorter than the duration of normal tests.

The acceleration factor (k_acc_) at increased supply voltage is calculated according to [18] using Formula (7):

(7) $$k_{acc}=\exp\cdot \alpha \cdot (U_{f}-U_{0}),$$

where *U*_f_, *U*_0_—value of supply voltage in boost and normal modes, respectively; *α*—model constant equal to 0.1 V^−1^ according to [18].

For microcircuits of TPS54332 series, the following UI mode at accelerated tests on reliability and MTBF at increased supply voltage is chosen: *U*_f_ = 33 V, *U*_0_ = 24 V, at *K*_U_ = 2.46. In this case, the current value was 16 mA, and the switching frequency was 100 Hz.

At current forcing, the acceleration factor is calculated by Formula (8) according to [16].

(8) $$k_{acc}={\left(\frac{J_{f}}{J_{0}}\right)}^{n},$$

where *J*_f_ and *J*_0_ are the values of current density through the product element in forced and normal modes, respectively; n is the model constant.

When calculating the acceleration coefficient for current forcing in a wide range of current densities in which n has different values, the total acceleration coefficient is calculated as the product of the acceleration coefficients in different current ranges.

For thermally activated degradation processes, the acceleration (forcing) coefficient is calculated by Formula (9) [16].

(9) $$k_{acc}=\exp\left[\frac{E_{aj}}{k}\left(\frac{1}{T_{0}}-\frac{1}{T_{1}}\right)\right],$$

where *k* is the Boltzmann constant (8.617 × 10^−5^ eV/˚K); *T*_0_ and *T*_1_ are the transition temperatures of normal and forced modes; and *E_aj_* is the activation energy of the *j*-th degradation process. Microcircuits have a complex degradation process consisting of several cumulative degradation processes with different activation energies. Due to the fact that the degradation processes under consideration have different activation energies, with any change in the thermal load, the activation energy during the degradation process of microcircuits is experimentally determined.

Let us calculate the acceleration coefficient for three values of temperatures (25, 65, 85) °C using Formula (9). The results of calculations of acceleration coefficients are presented in Table 4. If the processes have no thermal component, the acceleration factor is assumed to be equal to one.

**Figure 10:**
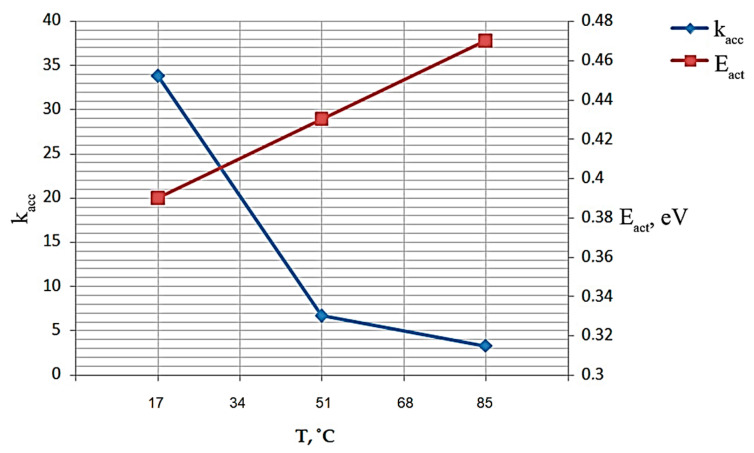
Dependence of acceleration coefficient and activation energy on temperature.

Figure 10 shows the dependencies of the acceleration coefficient and activation energy on the medium temperature.

Figure 10 shows that the activation energy increases linearly with increasing temperature. The acceleration coefficient, on the contrary, decreases.

The acceleration factor K_y_ for the selected accelerated mode (T, U, J) compared to the normal mode (T_0_, U_0_, J_0_) is calculated depending on the available data on the relative distribution of failure mechanisms in the total failure flow. Based on the conducted research, the existing methodology for accelerated testing at elevated temperature is validated.

## 6. Conclusions

We tested commercially produced ICs that are used in large volumes in power supplies. It was the large volumes of power supplies produced on the basis of the tested ICs that determined the importance of this work. Therefore, the authors have conducted reliability studies and lifetime studies of this IC. Experimental rejection tests were carried out, with subsequent analysis of the chip samples.

As a result of the work done, rejection tests of samples of the TPS54332 microcircuit under study were carried out. Experimental fail-safe tests were carried out with subsequent analysis of TPS54332 microcircuit samples by the controlled parameter quiescent current. On the basis of the obtained experimental values, the values of activation energy and acceleration coefficient at different temperature regimes were calculated. Dependences of activation energy and acceleration coefficient on temperature were plotted, which show that activation energy linearly increases with increasing temperature, while the acceleration coefficient, on the contrary, decreases. It was also found that the value of the calculated activation energy is 0.1 eV less than the value of the activation energy specified by the chip manufacturer (Texas Instruments, Dallas, TX, USA). The acceleration coefficient for different temperatures (25, 65, 85) °C was determined.

On the basis of the research conducted on TPS54332 chip tests, the existing methodology of accelerated tests at elevated temperatures with a modified acceleration coefficient is confirmed to be valid for application to this chip. In the future, we plan to investigate the degradation of IC parameters during accelerated failure tests with the application of the calculated acceleration coefficient.

## Figures and Tables

**Table 1:** Electrical parameters of the microcircuit at acceptance and delivery.

Parameter Name, Measurement Unit, Measurement Mode	Parameter Letter Designation	Parameter Norm		Ambient Temperature (Enclosure), °C
		Not Less Than	Not More Than	
Quiescent current, mA, at U_bx =_ 24 V, U_ec_ = 2 V	I_pot_	-	5.4	25 ± 10
		-	9.0	−(60 ± 3); 85 ± 3
Standby quiescent current, mA, at U_bx_ = 24 V, U_crb_ = 0 V	I_pot.off_	-	0.9	25 ± 10
		-	1.35	−(60 ± 3); 85 ± 3
Output current limit of the microcircuit, A, at U_bx_ = 24 V, U_v_ = 5 V	I_vv2_	0.55	-	25 ± 10
		0.33	-	−(60 ± 3); 85 ± 3
Open key resistance, Ohm	R_otk2_	-	0.9	25 ± 10
		-	1.35	−(60 ± 3); 85 ± 3
Frequency of internal oscillator, kHz, at U_bx_ = 24 V, U_out_ = 5 V	f_g_	160	360	25 ± 10
		100	400	−(60 ± 3); 85 ± 3
Instability of the set output voltage over the entire input voltage range, mV, at U_out_ = 5 V, I_out_ = 0.1 A	∆U_u_	-	200	25 ± 10
		-	250	−(60 ± 3); 85 ± 3
Instability of the set output voltage over the whole range of load currents, mV, at U_bx_ = 24 V, U_v_ = 5 V	∆U_i_	-	400	25 ± 10
		-	450	−(60 ± 3); 85 ± 3

**Table 2:** Measured values of quiescent current at different dwell times during the test process.

			T_out_, h			
96	168	240	500	1000	2000	3000
			I_quiescent_, mA			
1.43	1.57	1.83	2.09	1.27	1.51	1.5
1.57	1.75	1.72	2.04	1.83	1.83	1.27
1.89	1.43	1.51	1.86	1.67	1.43	1.57
1.51	1.88	1.57	1.8	1.43	1.43	1.27
1.42	1.27	1.83	1.85	1.51	1.51	1.67
1.43	1.58	2.42	2.27	2.31	1.67	1.57
1.51	1.27	1.49	2.3	1.67	1.58	2.47
1.83	1.88	1.57	2.78	1.51	1.51	1.27
2.37	2.44	1.57	3.32	1.57	1.75	2.67
1.49	5.57	1.5	4.77	1.5	3.19	2.57

**Table 3:** Activation energy of the chip.

			T, °C		
	25	65	85	115	125
E_a_, eV	0.39	0.43	0.47	0.5	0.52

**Table 4:** Value of acceleration factor.

		T, °C	
	25	65	85
k_acc_	33.8	6.7	3.25

## Data Availability

The data presented in this study are available from the corresponding authors upon reasonable request.
